# Obesity and disease activity in juvenile idiopathic arthritis

**DOI:** 10.1186/1546-0096-10-3

**Published:** 2012-01-12

**Authors:** Christina F Pelajo, Jorge M Lopez-Benitez, Laurie C Miller

**Affiliations:** 1 Department of Pediatric Rheumatology, Floating Hospital for Children at Tufts Medical Center, 800 Washington St, Box #190 Boston, MA 02111

**Keywords:** Obesity, Overweight, Arthritis, Juvenile Rheumatoid, Rheumatology, Child, Inflammation

## Abstract

**Background:**

Children with physical disabilities may have an increased risk for obesity and obesity might be a risk factor for inflammatory arthritis. The aims of this study were: to determine the prevalence of obesity in children and adolescents with juvenile idiopathic arthritis (JIA), and to examine the association between obesity and disease activity in this population.

**Findings:**

A cross-sectional analysis of all patients with JIA attending a pediatric rheumatology clinic, between October 2009 and September 2010, was performed. A linear regression model was used to explore the association between obesity and disease activity in patients with JIA. A total of 154 subjects were included in the analysis; median age was 10.6 years, 61% were female, and 88% were white. Obesity was found in 18%, 12% were overweight, and 3% were underweight. There was no association between obesity and JADAS-27 (Juvenile Arthritis Disease Activity Score 27), physician's assessment of disease activity, parent's assessment of child's well-being, erythrocyte sedimentation rate, number of active joints, or C-reactive protein (p-value range 0.10 to 0.95).

**Conclusions:**

Although 18% of patients with JIA were obese, we did not find an association between obesity and disease activity. As obesity confers an additional health risk in children with arthritis, addressing this co-morbidity should be a health priority in patients with JIA. Future studies are necessary to further explore potential associations between obesity, development of JIA, and disease activity.

## Background

Obesity is of epidemic proportions throughout the world [1-3]. The prevalence estimates are even more alarming in developed countries [1]. For example, recent data from the CDC shows that 20,7% of schoolchildren in New York City are obese [4-6].

Children with physical disabilities may have an increased risk for obesity and the rates of overweight and obesity in patients with juvenile idiopathic arthritis (JIA) have ranged from 5 to 23% [7-10]. On the other hand, it is possible that obesity might be a risk factor for inflammatory arthritis. In adults, the odds of having arthritis, other than osteoarthritis, are 4.3 higher among the obese population [10]. A possible relationship between adipose tissue and inflammatory arthritis is through the role of adipokines. All major adipokines (leptin, adiponectin, visfatin, and resistin) bear immune-modulatory properties and evidence points to involvement in the pathophysiology of rheumatoid arthritis (RA) [11]. Studies have shown an increased level of leptin (a pro-inflammatory adipokine) in the serum and in the synovial fluid of RA patients [11]. There is also evidence showing that adipocytes produce cytokines like TNF α, IL-1 and IL-6. Besides, pathophysiology of RA involves T cells, mast cells, and macrophages, which are common to adipose tissue pathophysiology as well [11].

Obesity has also been associated with worse disease activity in adults with RA [12,13]. Correlations between leptin and disease markers like DAS-28, erythrocyte sedimentation rate (ESR), and the number of tender joints have been shown in patients with longer disease duration [11].

While obesity may be a risk factor for, or a consequence of arthritis, it is unknown whether it is associated with the severity of disease activity in patients with JIA. The objectives of this study were to determine the prevalence of obesity in patients with JIA, and to examine the association between obesity and disease activity in this population.

## Methods

A database was created of all patients with JIA attending a single pediatric rheumatology clinic in Boston, between October/2009 and September/2010 who had blood drawn for routine clinical monitoring. In a period of 1 year virtually every patient with JIA has blood drawn at least once. From all eligible patients, only 1 declined to be included in the database. Patients who had taken corticosteroids in the previous 3 months, who were not English-speaking, who had any infections during the 2 weeks before the appointment, and who had concurrent medical problems (diabetes type 1, inflammatory bowel disease, celiac disease, and immunodeficiency) were excluded. Body mass index (BMI), disease activity scores, and other demographic and clinical information were retrieved from this database. This project was approved by the Institutional Review Board at Tufts Medical Center.

Disease activity was measured on the same visit of the blood tests, using a validated score, JADAS- 27 (Juvenile Arthritis Disease Activity Score 27) [14]. This score includes four measures: physician global assessment of disease activity using a visual analog scale (VAS) [12], parent global assessment of child's well-being determined by a VAS, count of joints with active disease (evaluating 27 joints), and ESR. ESR is normalized to a score ranging from 0 to 10, by the formula (ESR-20)/10. JADAS-27 is calculated as the simple linear sum of the scores of its 4 components, which yields a total score of 0-57, with higher scores associated with worse disease activity.

BMI values were plotted using the 2000 CDC growth charts (BMI for age) [5], and BMI percentiles were determined. Children were classified as obese if their BMI was ≥ 95^th ^percentile, overweight if their BMI was between the 85^th ^and 94^th ^percentile, and healthy weight if their BMI was between the 5^th ^and the 84^th ^percentiles [4,6]. Prevalence of underweight (BMI < 5^th ^percentile) was also determined, however, there was a very small number of patients in this category, hence they were grouped under healthy weight for statistical analysis purposes.

Basic demographic and health information about study subjects was also collected including the following: age, gender, ethnicity, JIA subtype, time interval since disease onset, current medications, and C-reactive protein value (CRP). Medications used for JIA treatment were recorded (non-steroidal anti-inflammatory drugs, methotrexate, biologic drugs), as well as the duration of use of each medication. Subjects were only considered to be on methotrexate or a biologic drug if the duration of use was ≥ 2 months.

### Statistical analysis

The prevalence of obesity was determined. Linear regression was used to examine the association of obesity with JADAS-27, physician VAS, parent VAS, number of active joints, and CRP levels. Logistic regression was used to examine the association between obesity and ESR (dichotomized to normal, if ≤20, or abnormal). A multivariable linear regression model was developed to analyze the association between obesity and the outcome JADAS-27. The model was adjusted for potential confounders, identified *a priori*, such as age, gender, JIA subtype, ethnicity, medications (none, non-steroidal anti-inflammatory drugs, or immunosuppressants - methotrexate and/or biologics), and time since disease onset.

JMP version 9 (SAS Institute Inc., Cary, NC) was used for data analyses. All tests were two-sided and statistical significance was defined as a two-sided p-value < 0.05.

## Results

A total of 154 subjects were included in the analyses. Subjects' characteristics are shown by BMI category in Table 1. There were no differences in patients' characteristics by BMI category. The median age of participants' was 10.6 years (± 4.5, range 2-19), 61% were female, and 88% were white.

**Table 1:** Demographic characteristics of patients by BMI category

	Obesity (n = 28)	Overweight (n = 19)	Healthy weight (n = 107)	P value
**Age **(median, IQR^a^)	9, 6 - 13	10, 6 - 13	11, 7 - 15	0.23

**Females **% (N)	54 (15)	58 (11)	64 (68)	0.60

**Ethnicity **% (N)				0.42
Caucasian	93 (26)	84 (16)	87 (93)	
African American	0	0	4 (4)	
Hispanic	7 (2)	16 (3)	5 (6)	
Asian	0	0	4 (4)	

**JIA subtype **% (N)				0.27
Oligoarticular	36 (10)	53 (10)	47 (51)	
Rheumatoid factor-negative polyarticular	21 (6)	10 (2)	23 (25)	
Rheumatoid factor-positive polyarticular	0	0	4 (4)	
Systemic-onset	4 (1)	5 (1)	2 (2)	
Enthesitis-related arthritis	18 (5)	26 (5)	18 (19)	
Psoriatic arthritis	21 (6)	5 (1)	6 (6)	

**Time since JIA onset **in months (median, IQR^a^)	28, 7 - 66	34, 4 - 61	25, 5 - 68	0.90

**Medications **% (N)				
Non-steroidal anti-inflammatory drugs	50 (14)	58 (11)	49 (52)	0.75
Methotrexate	21 (6)	10 (2)	19 (20)	0.62
Biologics	14 (4)	16 (3)	17 (18)	0.95
Intra-articular steroids	4 (1)	0	5 (5)	0.62
None	36 (10)	37 (7)	31 (33)	0.81
**Time on medications **in months (mean ± SD)				
Non-steroidal anti-inflammatory drugs	13 ± 34	6 ± 9	10 ± 22	0.88
Methotrexate	13 ± 42	2 ± 6	5 ± 15	0.62
Biologics	4 ± 10	4 ± 12	3 ± 9	0.98

^a^IQR: interquartile range

Median BMI was 19 and median BMI percentile was 67 (interquartile range 41-88). Eighteen percent of the patients (n = 28) were obese, 12% (n = 19) were overweight, and 3% (n = 4) were underweight.

The score of JIA disease activity, JADAS-27, had a median value of 5.2 (range 0-30.7). The components of JADAS-27 and its individual scores are described by BMI category in Table 2.

**Table 2:** Disease activity variables by BMI category


	**Obesity** **(n = 28)**	**Overweight** **(n = 19)**	**Healthy weight** **(n = 107)**	**P value**

**C-reactive protein **mg/L (mean ± SD)	2 ± 2	1 ± 2	3 ± 9	0.12

**Parent VAS **(median, IQR^a^)	2.2, 0.8 - 4.6	1.5, 0.6 - 3.2	1.3, 0.5 - 2.6	0.24

**Physician VAS **(median, IQR^a^)	1.3, 0 - 2.7	0.9, 0 - 2.2	1.3, 0.5 - 2.6	0.42

**ESR **in mm/hr (median, IQR^a^)	14, 9 - 21	14, 10 - 20	10, 8 - 16	0.10

**Joint count **(median, IQR^a^)	1.5, 0 - 4	1, 0 - 3	2, 0 - 4	0.08

**JADAS - 27 **(median, IQR^a^)	5.6, 2.2 - 10	3.4, 1.8 - 8	5.2, 2.2 - 10.5	0.44

^a^IQR: interquartile range

There was no association between obesity and JADAS-27 in the univariable linear regression (beta coefficient = 0.04; 95%CI = -1.19, 1.27; p = 0.95), neither between obesity and physician's VAS (beta coefficient = 0.08; 95%CI = -0.28, 0.44; p = 0.66), parent's VAS (beta coefficient = -0.35; 95%CI = -0.77, 0.07; p = 0.10), ESR (OR = 0.53; 95%CI = 0.21, 1.41; p = 0.18), number of active joints (beta coefficient = 0.22; 95%CI = -0.39, 0.83; p = 0.48), or CRP (beta coefficient = 0.33; 95%CI = -1.19, 1.85; p = 0.67). In the multivariable linear regression model, obesity did not have a significant effect on JADAS-27 scores (beta coefficient = -0.12; 95%CI = -1.31, 1.07; p = 0.84), after adjusting for age, gender, JIA subtype, medication use, time since disease onset, and ethnicity.

## Discussion

Despite a high rate of obesity in this sample of patients with JIA, there was no association between obesity and disease activity, measured by several variables.

Nearly one-fifth of children and adolescents in our sample were obese. This alarming rate is similar to the rates of obesity previously reported in otherwise healthy children, as well as in children with JIA [6-8].

Obesity has been associated with worse disease activity in RA patients [12,13]. However, we did not find this association in our sample of JIA patients. Although there are no other published studies examining the association between obesity and disease activity in JIA, a Brazilian study also failed to find any difference in median BMI percentiles between patients with active and inactive disease [9]. In a cross-sectional study of RA patients, an association between Health Assessment Questionnaire results and BMI was found in the initial analysis, but once physical activity was introduced as a confounder, the association was lost. Hence it is possible that the effects of inflammation on body composition were mediated via physical (in)activity [15].

Even though body fat and obesity have been associated with increased inflammatory markers (ESR and CRP [12,16,17]), we did not find this association in our sample of JIA patients. Similarly, in other studies of children with arthritis, ESR and CRP did not correlate with BMI [9,18]. Also studies involving RA patients failed to show an association between CRP and obesity or BMI [13,15,19].

Interestingly, BMI increase has been associated with the use of anti-TNF agents [20,21]. Although the role of anti-TNF drugs on weight regulation needs to be confirmed, one hypothesis to explain these findings is that inhibition of TNF-α driven hypermetabolism results in weight gain [22]. However, we did not find any differences on the rate of use of medications in the different BMI categories.

Our study had several limitations, such as the cross-sectional design, and the relatively small sample size, which could be responsible for inadequate statistical power. We did not have information on physical activity, socioeconomic status, and parents' education level and BMI, all of which have been previously associated with obesity.

However, to the best of our knowledge, this is the first study to investigate the association between obesity and disease activity in patients with JIA.

In conclusion, although 18% of JIA patients in this study were obese, we did not find an association between obesity and disease activity. As obesity confers an additional health risk in children with arthritis, addressing this co-morbidity should be a health priority in patients with JIA. Future studies are necessary to further explore potential associations between obesity, development of JIA, and disease activity.

## List of abbreviations

JIA: juvenile idiopathic arthritis; RA: rheumatoid arthritis; BMI: Body mass index; JADAS- 27: Juvenile Arthritis Disease Activity Score 27; VAS: visual analog scale; ESR: erythrocyte sedimentation rate; CRP: C-reactive protein value.

## Competing interests

The authors declare that they have no competing interests.

## Authors' contributions

CFP participated on the conception and design of the study, acquisition of data, analysis and interpretation of data, as well as drafting the manuscript. JML participated in the design of the study, data collection, interpretation of data, and revision of the manuscript. LCM participated in the design of the study, data collection, interpretation of data, and revision of the manuscript. All authors read and approved the final manuscript.
